# Impact of saccharides on the drying kinetics of agarose gels measured by *in-situ* interferometry

**DOI:** 10.1038/srep41185

**Published:** 2017-01-23

**Authors:** Bosi Mao, Thibaut Divoux, Patrick Snabre

**Affiliations:** 1 Centre de Recherche Paul Pascal, CNRS UPR 8641 - 115 avenue Dr. Schweitzer, 33600 Pessac, France; 2 MultiScale Material Science for Energy and Environment, UMI 3466, CNRS-MIT, 77 Massachusetts Avenue, Cambridge, Massachusetts 02139, USA

## Abstract

Agarose gels are viscoelastic soft solids that display a porous microstructure filled with water at 90% w/w or more. Despite an extensive use in food industry and microbiology, little is known about the drying kinetics of such squishy solids, which suffers from a lack of time-resolved local measurements. Moreover, only scattered empirical observations are available on the role of the gel composition on the drying kinetics. Here we study by *in-situ* interferometry the drying of agarose gels of various compositions cast in Petri dishes. The gel thinning is associated with the displacement of interference fringes that are analyzed using an efficient spatiotemporal filtering method, which allows us to assess local thinning rates as low as 10 nm/s with high accuracy. The gel thinning rate measured at the center of the dish appears as a robust observable to quantify the role of additives on the gel drying kinetics and compare the drying speed of agarose gels loaded with various non-gelling saccharides of increasing molecular weights. Our work shows that saccharides systematically decrease the agarose gel thinning rate up to a factor two, and exemplifies interferometry as a powerful tool to quantify the impact of additives on the drying kinetics of polymer gels.

Hydrogels consist in a wide variety of soft viscoelastic solids that are commonly encountered in nature, as exemplified by hagfish slime^1^, and lie at the core of numerous industrial applications such as scaffolds for tissue engineering^2^, growth culture media^3^,^4^, controlled drug release^5^,^6^, etc. Hydrogels are mainly composed of water and contain only a few percent in mass of natural or synthetic polymers that are linked together either by covalent bonds, or by physical interactions such as hydrogen bonds, or dipole-dipole interactions, etc.^7^,^8^. In both cases, polymers form a fibrous-like, sample-spanning network that is responsible for the gel viscoelastic behavior under low external strain^9^. Moreover, due to their fibrous structure composed of interconnected nonlinear springs, hydrogels experience a pronounced hardening under larger deformations^10^,^11^,^12^,^13^, up to the formation of macroscopic fractures, which are characteristic of a brittle rupture scenario^14^,^15^.

Being mainly composed of water, polymer gels are highly sensitive to water loss through evaporation and stress-induced solvent release^16^. The drying of polymeric gels has been quantified by macroscopic observations^17^, weighing^18^, and more local investigation techniques such as small angle neutron scattering^19^, fluorescence spectroscopy^20^, and interferometry^21^,^22^. The goal of previous studies was to set the basis of a thermodynamics of swelling and shrinking, and among other things to test Li and Tanaka predictions for the drying kinetics of crosslinked polymeric networks^23^. Previous experiments mainly consisted in monitoring the shrinkage of a disc-shaped gel with free boundary conditions. In the present work, we tackle the case of hydrogels cast in a cylindrical dish. The weak adhesion between the gel and the lateral wall of the dish allows for the shrinkage to be unidirectional along the vertical axis and the gel to remain in contact with the dish lateral wall. We thus can focus on the vertical thinning rate of the gel.

Here, we choose to work with agarose, which is a natural neutral polymer extracted from a red marine algae and composed of disaccharide units^8^,^24^,^25^. Agarose is the gelling agent of culture media commercialized in Petri dishes^26^. These media are routinely incubated at constant temperature to monitor the potential growth of bacterial colonies. The gel experiences drying during that process, which leads to the gel shrinkage and often to the gel detachment from the lateral wall of the Petri dish^27^, invalidating bacterial counts. Moreover, such culture media contain numerous additives, including non-gelling saccharides such as agaropectin, sucrose, etc. that affect the water-holding capacity of the gel and potentially impact the gel drying kinetics. Therefore, understanding the drying kinetics of agarose gels and the influence of minute amounts of additives upon the gel thinning rate is of key practical importance.

Here we use interferometry as a tool to measure with high accuracy the local thinning rate 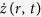 of agarose gels. The drying dynamics of a gel cast in a plate exhibits three phases. During most of the drying process the gel thins at constant speed, then experiences a sudden acceleration before coming to a complete stop. Experiments conducted at different positions *r*_0_ along the dish radius *r* show that the gel thinning rate remains constant except at the very end of the drying process and follows the same overall scenario. We further demonstrate that the thinning rate 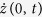 measured at the center of the dish is independent of the gel thickness and diameter, and as such can be used as a robust observable to compare the thinning rate of agarose gels loaded with minute amounts of additives. In particular, we compare the thinning rates of gels made either of agarose or agar, i.e., mixture of agarose and agaropectin. We show that for equal contents in agarose (larger than 0.5% w/w), agar gels thin 40% slower than pure agarose gels due to the presence of agaropectin. More generally, we show that the thinning rate of agarose gels is systematically reduced by addition of minute amounts (<0.5% w/w) of non-gelling saccharides such as glucose, dextran, guar gum or xanthan gum. Finally, we observe that the gel thinning rate poorly depends on the molecular weight of the additive and is mainly a function of the additive concentration.

Up to now non-gelling saccharides such as sucrose, glucose, maltose, xanthan gum, etc. have been shown to impact the formation of agarose gels^28^,^29^,^30^ and their mechanical properties^31^,^32^,^33^ when introduced in large amounts (5% w/w or higher). An increased mass of non-gelling polysaccharides leads to (*i*) larger gel elastic modulus, (*ii*) larger strain and stress at failure and (*iii*) lower water release under external load^34^. However, the impact of non-gelling saccharides on the drying kinetics of agarose-based gels remains an open issue that is the topic of the present study. Furthermore, we choose here to focus on low additives content (0.5% w/w or lower). In that range of concentrations the linear mechanical properties of agarose gel remain unaffected by the additives, which allows us to disentangle the water-holding capacity induced by the additives from their impact on the gel elastic properties.

## Results

### Michelson interferometer

**Figure 1:**
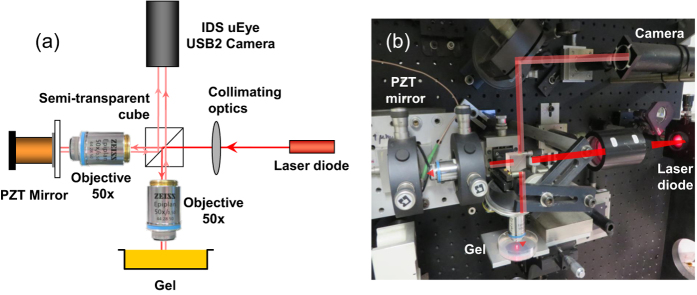
(**a**) Sketch of the experimental setup that consists in a Michelson Interferometer operated in reflection mode. (**b**) Photo of the real experimental setup. The scale is set by the diameter of the Petri dish of 50 mm. The experimental setup is placed in a box that can be closed with thick curtains to limit air convection that may otherwise artifically increase the gel thinning rate.

**Figure 2:**
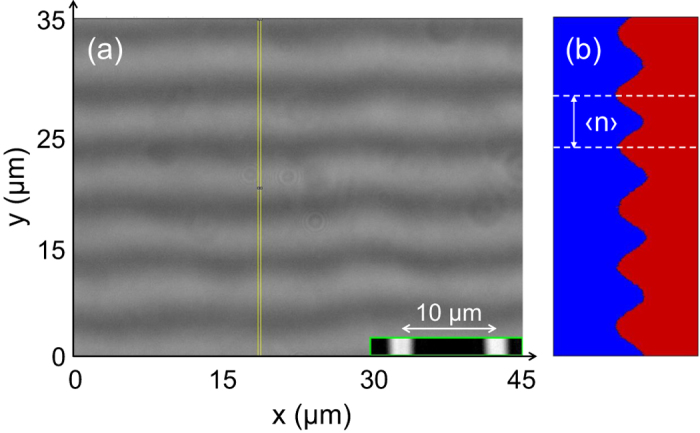
(**a**) Typical instantaneous interference pattern overlayed on the image of the smooth gel surface (800 pixels × 600 pixels). Inset: image of a 10 *μ*m grid from a Leitz standard stage micrometer. (**b**) Projection along the *y*-axis of the gray levels of all the pixels enclosed in the thin yellow vertical rectangle selection pictured in (**a**). The white dashed lines highlight the average distance 〈*n*〉 between two successive dark fringes. Experiment conducted on a 1.5% agar gel cast in a plastic Petri dish made of PS.

A gel cast in a Petri dish made of glass or plastic (polystyrene crystal, denoted as PS) as described in the Method section is left to dry at T = (25.0 ± 0.5) °C unless otherwise specified. The height variations of the gel are monitored by means of a Michelson interferometer operated in reflection mode. The principle of a Michelson Interferometer pictured in Fig. 1 is to split a partially coherent incident beam (red laser diode M625L3 from Thorlabs with a wavelength *λ* = 625 nm and a coherence length of about 50 *μ*m) into two beams: a reference beam reflected by a PZT mirror and a second beam reflected by the surface of the gel. A collimating optics produces a parallel beam splitted with a semi-transparent cube. A flat field planachromat objective without immersion (Zeiss LD Epiplan 50× with an extra long working distance of about 9.1 mm and a numerical aperture of 0.5) forms the image of the gel in the focal plane of the camera. A second identical objective focuses the reference wave on the mirror. The mirror of the reference arm is mounted on a piezoelectric tube (Unidex 11, Aerotech) that allows us to adjust the optical phase difference between the reference and the signal arms with a nanometric accuracy so that the two waves interfere at the level of the sensitive surface of the camera (USB2 monochrome IDS uEye camera, UI124 × SE-M) and produce a set of almost parallel and contrasted interference fringes overlayed on the image of the smooth gel surface. A typical image recorded at the gel surface is pictured in Fig. 2(a).

### Images analysis: spatiotemporal filtering method

In order to access the thinning rate of the gel from Fig. 2(a), we use the following protocol. We first adjust the mirror orientation so that the interference fringes are horizontal (or vertical) with about 5 to 7 fringes within the field of view (45 *μ*m × 35 *μ*m) [Fig. 2(a)]. We then record a stack of 2400 TIF images during ten minutes at a frequency *f* = 4 frames per second. The contrast of fringes remains almost unaltered during that time since the gel thins by about 8 *μ*m to 15 *μ*m, which is much less than the coherence length of the source (of about 50 *μ*m). The average number 〈*n*〉 of pixels between two fringes, which corresponds to a half wavelength in terms of height differences that give rise to interferences, is determined through a projection of the gray level of all the pixels in the thin rectangular region of interest (ROI) pictured in yellow in Fig. 2(a,b)].

**Figure 3:**
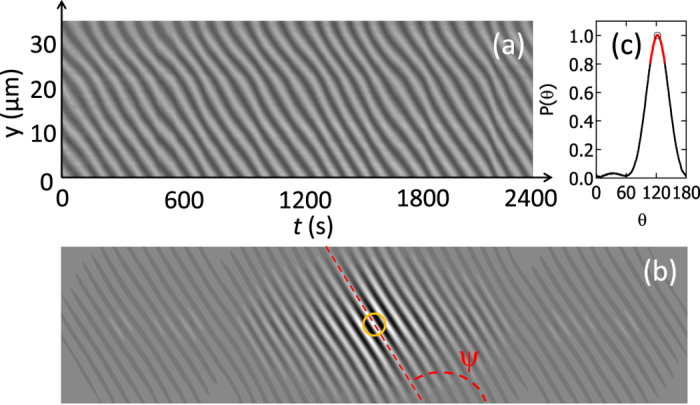
(**a**) Spatiotemporal diagram *T(y, t*) that results from projection over 20 min of the gray levels of all the pixels within the thin yellow rectangle pictured in Fig. 2(a) and oriented in the direction of the fringes displacement. (**b**) Auto-correlation function 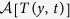 of the spatiotemporal diagram *T(y, t*) pictured in (**a**). (**c**) Angular distribution *P(θ*) obtained from a radial integration of 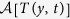 inside a circular ROI of 50 pixel radius that is centered on the origin of the autocorrelation image [yellow circle pictured in (**b**)]. The fit of the angular distribution *P(θ*) by a Lorentzian function over an angular sector of 30° around the maximum [red curve in (**c**)] allows us to determine the tilt angle Ψ = 121.1° of the ridge line [red dashed line in (**b**)], and to compute the gel thinning rate *v* = 15.7 nm/s averaged over 20 min. Experiment conducted at the center of a 1.5% w/w agar gel cast in a plastic Petri dish made of PS.

The subsequent analysis of the complete stack of images allows us to compute the average thinning rate 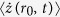 of the gel over 10 minutes (or shorter duration if need be), where *r*_0_ denotes the distance between the center of the gel and the position where the beams impacts the gel, and *t* labels the time since the start of the drying experiment. We also compute the standard deviation of the thinning rate 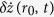, representative of both temporal fluctuations in the drying dynamics and any lateral sliding motion of the gel on the bottom wall of the dish. The analysis is performed using a spatiotemporal filtering method based on the idea that detecting motion is equivalent to extracting an orientation inside a correlation image. The temporal projection of the gray levels of all the pixels within the thin yellow vertical ROI pictured in Fig. 2(a) and oriented in the exact direction of the fringes displacement produces a spatiotemporal diagram *T(y, t*) featuring a pattern of oblique lines [Fig. 3(a)]. The average tilt angle Ψ of the oblique lines with respect to the horizontal time axis is directly related to the average thinning rate of the gel at the position *r*_0_ through the following relation:


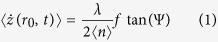


To determine the tilt angle Ψ and deduce the average thinning rate, we compute the auto-correlation 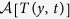 of the spatiotemporal diagram *T(y, t*) using a Discrete Fast Hartley Transform, which minimizes the computer memory required and the number of arithmetic operations compared to the traditional Fast Fourrier Transform Fourier Transform^35^,^36^. The auto-correlation shows a sharp line (or ridge line) in the center with the same average orientation Ψ as the oblique lines in the spatiotemporal diagram *T(y, t*) [Fig. 3(b)]. A radial integration of 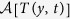 inside a circular ROI centered on the origin of the autocorrelation image [radius p = 50 pixels, yellow circle in Fig. 3(b)] provides the angular distribution *P(θ*), which is well fitted by a Lorentzian function [Fig. 3(c)] and gives the tilt angle Ψ of the ridge line [red dashed line in Fig. 3(b)]. We shall emphasize that unlike traditional PIV methods, which cross correlate two consecutive interrogation regions, the spatiotemporal methods analyze a continuous space-time window and not a discrete displacement, which offers a higher accuracy to determine the thinning rate of the gel in a shorter computing time. For an optimal precision, the method yet assumes a constant brightness of both the moving fringes and the gel image. Finally, note that the interferometer and the gel are placed in a box closed by thick curtains for the whole duration of each experiment to limit as much as possible changes in humidity and air convection, which otherwise would affect the drying process.

### Local vs. global measurements

**Figure 4:**
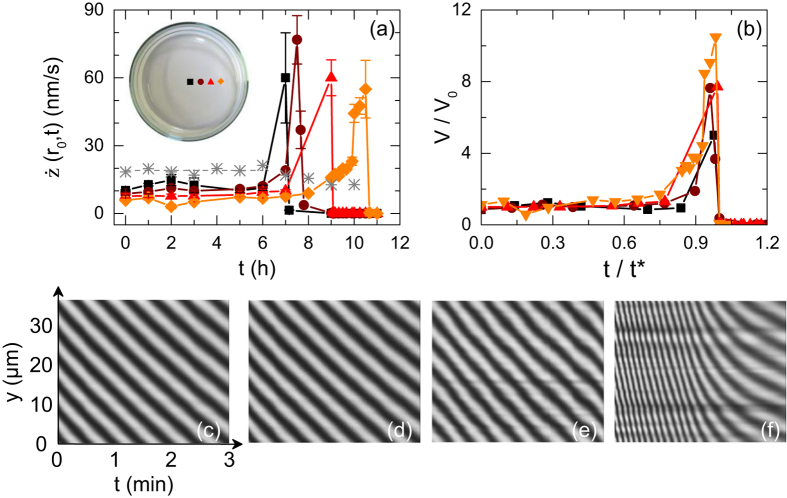
(**a**) Local thinning rate 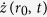 vs time *t* for a 1.5% w/w agar gel (*e* = 1 mm) at different locations *r*_0_ from the center of the plate (diameter 2*R*): *r*_0_/*R* = 0 (■), 0.2 (•), 0.4 (▲) and 0.6 (♦). The stars (*) stand for independent mass-loss measurements performed with a precision scale indicating an average global thinning rate of 19.1 ± 0.5 nm/s over the first 6 hours (horizontal dashed line). (**b**) Same data as in (**a**) where the local thinning rate for each data set has been normalized by the local value *V*_0_ averaged over the first 6 hours and the time *t* has been normalized by the time t^*^(*r*_0_) associated with the largest thinning rate reached at a radial position *r*_0_ during the drying process. (**c**–**f**) Spatio-temporal diagrams of the fringe displacement recorded over a period of 10 min during the drying process at the center of the gel and at different times *t* = 0, 3, 6 and 7 h. The auto-correlation of each of these patterns provides the average thinning rate: 10.2 ± 0.8 nm/s (**c**), 12.5 ± 1.0 nm/s (**d**), 11.3 ± 0.9 nm/s (**e**) and 58 ± 20 nm/s (**f**). These values are used to build the curve pictured as (■) in (**a**). Experiments conducted in a Petri dish made of glass at *T* = (21.0 ± 0.5) °C.

**Figure 5:**
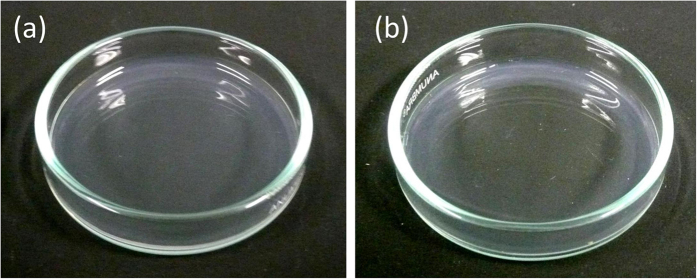
Pictures of an agar gel of average thickness 1 mm, cast in a Petri dish of 50 mm diameter made of glass: (**a**) prior to the drying experiment, and (**b**) 8 hours after the start of the drying experiment at T = (21.0 ± 0.5) °C. The complete drying occurs first at the center of the dish, which suggests a slightly larger thinning rate of the gel at the center of the dish, as quantified in Fig. 4.

We first report measurements at *T* = (21.0 ± 0.5) °C during the drying process of 1.5% w/w agar gel of 1 mm thick and cast in a Petri dish of diameter 50 mm made of glass. The thinning rate averaged periodically over a duration of 10 min is measured as a function of time at the center of the dish and at three other locations *r*_0_ along the dish radius [*r*_0_/*R* = 0, 0.2, 0.4, and 0.6 in Fig. 4(a)]. During the first 6 hours, the gel thins at constant speed at the center of the dish. For *t* ≥ 6 h the thinning-rate increases abruptly, goes through a maximum and stops as the drying process ends. A similar scenario is observed at the 3 other locations but shifted in time, which shows that the drying process is spatially heterogeneous: the gel thins slightly faster at the center of the dish. Indeed, the center region of the gel becomes dry first (see Fig. 5 for two images of the gel at different drying stages). Despite such an inhomogeneous drying process, the evolution of 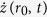 is robust for different radial positions *r*_0_ along the gel radius, as confirmed by the rescaling of the data on a single mastercurve [Fig. 4(b)].

Interestingly, the local average thinning rates measured by interferometry during the first 6 hours are almost two times smaller than the global thinning rate determined by independent mass-loss measurements [(*) in Fig. 4(a)]. Both local and global measurements are performed in the exact same conditions, which suggests that the water loss is more important than expected from the sole local measurements, which are limited to *r*_0_/*R* ≤ 0.6. Indeed, unfortunately, due to the lateral wall of the dish, one cannot approach the objective near the peripheral region of the sample and focus on the gel close from the wall. Direct observations of the dish from the side shows that the gel exhibits a curved meniscus of a few millimeter height in the vicinity of the lateral wall. Water evaporates most likely faster through that meniscus due to edge effects^37^, which accounts for the apparent discrepancy between local and global measurements. Furthermore, the maximum thinning rate of about 60 nm/s reported in Fig. 4(a) corresponds to the passage under the observation region (*r*_0_) of the triple line that marks the frontier between the dry gel, the humid gel and air. Water evaporation is most likely enhanced at the very location of the triple line moving towards the lateral wall of the dish, which also accounts for the discrepancy between the local and global measurements.

In conclusion, we shall keep in mind from this first section that the interferometer makes it possible to monitor with a high accuracy the gel thinning rate averaged over short periods of time (typically 10 min) and its temporal evolution at various locations of the gel free surface. The thinning rate is maximum at the center of the dish and constant over the first 6 hours of the drying process. In the rest of the manuscript, we use the thinning rate 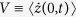 measured at the center of the dish and averaged over 10 minutes as a key observable independent of time to investigate the influence of the dish geometry on the drying process and quantitatively compare the thinning rate of agarose gels loaded with saccharides of various molecular weights.

### Role of the dish geometry on the thinning rate

**Figure 6:**
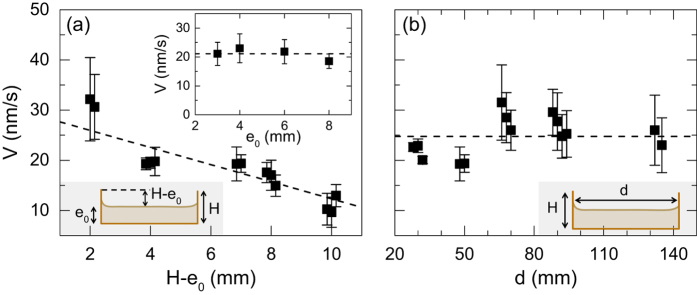
(**a**) Average thinning rate *V* of 1.5% w/w agar gels, measured at the center of the Petri dish vs. the height *H* − *e*_0_ of the lateral wall relative to the gel thickness, where *H* and *e*_0_ stand respectively for the height of the lateral wall of the Petri dish and the initial thickness of the gel (see the sketch in the lower inset). The dish height *H* is kept constant and the thickness of the gel is varied from *e*_0_ = 2 mm to 10 mm, such that *H* − *e*_0_ varies from 2 mm to 10 mm. For each value of *H* − *e*_0_, we perform three independent experiments on gels prepared anew. Each point corresponds to an average over 10 minutes and the error bars represent the standard deviation computed over the same duration. The dashed line is the best linear fit of the data: *V* = (29.3 ± 2.3) − (1.7 ± 0.3)(*H* − *e*_0_). Inset: average thinning rate *V* of 1.5% agar gels measured at the center of the dish vs. the gel initial thickness *e*_0_. The relative height of the dish with respect to the gel thickness is kept constant for all the experiments: *H* − *e*_0_ = 4 mm. The dashed line stands for the average value: *V* = 21 ± 1 nm/s. All the experiments are performed at T = (25.0 ± 0.5) °C in a plastic Petri dish made of PS (*H* = 12 mm), except for the experiments at *H* − *e*_0_ = 7 mm reported in the main graph, which are performed in a dish made of glass (*H* = 11 mm). (**b**) Average thinning rate *V* of 1.5% agar gels measured at the center of the dish and averaged over 10 minutes vs. the dish diameter *d*. The relative height of the lateral wall compared to the gel thickness is kept constant *H* − *e*_0_ = 7 mm for all the experiments. For each value of the dish diameter *d*, we perform two to four independent experiments on gels prepared anew. Error bars correspond to the standard deviation of the thinning rate over 10 minutes. The horizontal dashed line stands for the average value: 〈*V*〉 = 24.7 nm/s. Experiments conducted at T = (25.0 ± 0.5) °C in Petri dishes made of glass.

We now aim at characterizing the influence of the geometrical characteristics of the Petri dish on the gel average thinning rate *V* measured at the center of the dish. First, we measure the average thinning rate of 1.5% w/w agar gels of various initial thicknesses *e*_0_ ranging from 2 mm to 10 mm, and cast in plastic Petri dishes made of PS (diameter of 50 mm and fixed height *H* = 12 mm). We can thus explore the effect of the height of the dish lateral wall relative to the gel initial thickness *e*_0_ measured prior to any drying. Thinning rates determined for various values of *H* − *e*_0_ at the center of the dish are reported in Fig. 6(a). The average thinning rate of the gel *V* decreases for increasing values of *H* − *e*_0_, the height of the lateral wall relative to the gel thickness. A higher lateral wall relative to the gel thickness delays the water evaporation by limiting the diffusion of water vapor above the gel free surface and creating a humid area, which leads to lower thinning rates. Furthermore, we perform a series of experiments on 1.5% w/w agar gels of various initial thicknesses *e*_0_ in Petri dishes of various heights *H*, while keeping constant the height *H* − *e*_0_ = 4 mm of the lateral wall relative to the gel thickness. The average thinning rate *V* of the gel is constant within errors bars for gels thicknesses ranging between 3 mm and 8 mm, showing that for a constant value of *H* − *e*_0_, the gel thickness does not influence the gel thinning rate [Inset in Fig. 6(a)]. The gel thinning rate at the center of the dish is thus mainly controlled by the water-vapor environment located above the gel free surface, and enclosed between the gel free surface and the maximal height of the dish lateral walls.

Second, we perform drying experiments in Petri dishes of different diameters, every other parameters kept constant (*H* − *e*_0_ = 7 mm). Thinning rates reported in Fig. 6(b) appear independent of the dish diameter *d* over the following range 30 ≤ *d* ≤ 135 mm, which confirms that the average thinning rate *V* measured at the center of the dish is a robust observable to characterize the drying of agarose gels. In the rest of the manuscript, both the dish diameter and the height of the lateral wall relative to the gel thickness are kept constant (*d* = 50 mm, *H* = 12 mm and *H* − *e*_0_ = 8 mm) so as to investigate the influence of the agarose concentration and the presence of supplemental non-gelling saccharides of increasing molecular weights on the gel thinning rate.

### Role of additives on the thinning-rate

#### Agarose vs. agar gels

**Figure 7:**
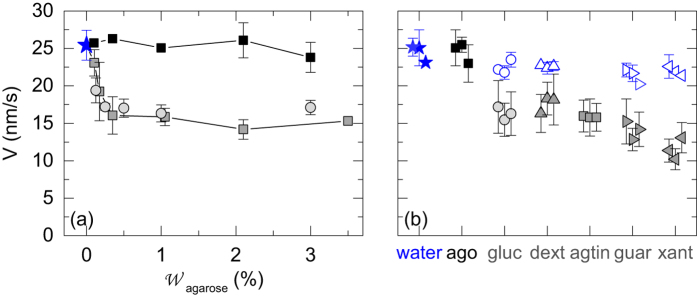
(**a**) Average thinning rate *V* determined at the center of the Petri dish for agarose gels (■), agar gels composed at 70% of agarose 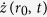 and agarose gels loaded with 0.43% w/w of glucose 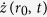, vs the concentration in agarose. The amount of saccharides (0.43% w/w) is chosen so that the ratio agarose/saccharide for all the samples is identical to the ratio agarose/agaropectin in the agar sample, which is 7:3. The blue star 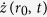 denotes the thinning rate of a water pool with the same volume as the agar(ose) gels and monitored in the exact same experimental condition (temperature 25 °C, and dish diameter 50 mm). Error bars correspond to average standard deviation associated with three independent measurements performed over 10 minutes each. Experiments conducted in a plastic Petri dish made of PS. (**b**) Average thinning rate *V* of a 1% w/w agarose gel (“ago”) and four different 1% w/w agarose gels loaded with 0.43% w/w of one of the following non-gelling saccharides: glucose (“gluc”, 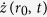), dextran (“dext”, 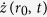), agaropectin (“agtin”, 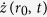), guar gum (“guar”, 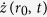) or xanthan gum (“xant”, 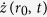). Note that the data for the gel loaded with agaropectin were measured directly on an agar gel - we did not mixed agaropectin and agarose. Finally, the blue open symbols (◦, ▵, ▹, ◃) stand for measurements performed on the aqueous solution of the corresponding saccharide, at the same concentration and without any agarose. Data are missing for an aqueous solution of agaropectin, which is not commercially available as a pure product. For each additive three independent experiments are performed on gels prepared anew. Error bars correspond to the standard deviation of the average thinning rate over the duration of the experiment (10 minutes). Experiments conducted at T = (25.0 ± 0.5) °C in a plastic Petri dish made of PS.

We now determine the average thinning rate *V* of agarose gels of various concentrations ranging from 0.125% to 3% w/w. Experiments are conducted at T = (25.0 ± 0.5) °C in a plastic Petri dish (PS) of 50 mm diameter. The results reported in Fig. 7(a) show that the thinning rate of agarose gels is constant and independent of the agarose content up to 3% w/w. Furthermore, the thinning rate is comparable to that of a water pool with the same initial volume as that of the gel [(★) in Fig. 7(a)]. This result suggests that agarose gels present negligible water-binding components and behave as passive sponges from which water evaporates freely. Furthermore, such result is in excellent agreement with independent compression experiments performed on gellan gels^38^ and quantitative NMR study of agarose gels^39^,^40^,^41^, which show that most of the water contained inside the gel is not bounded to the gel microstructure but free to diffuse.

We now compare the average thinning rate *V* of agarose and agar gels. Agar gels are composed of agarose and agaropectin, here in a ratio 7:3. Agaropectin is a charged polysaccharide structurally similar to agarose with the same repeating units, but with higher sulfate content^42^. As a consequence agaropectin does not gelify. The results of drying experiments of agar gels are reported in Fig. 7(a) as (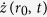). Over the whole range of agarose concentration explored, agar gels show systematically a smaller thinning rate than a gel that contains the same amount of agarose without any agaropectin. For concentrations in agarose lower than 0.4% w/w, the thinning rate of agar gel decreases for increasing agarose concentrations. Above 0.4% w/w of agarose the thinning rate is constant, independent of the agar(ose) content. This result, which holds true at lower temperature [see Fig. S3 in the Supplementary Information] strongly suggests that agaropectin presents water-binding sites that actively slow down the water evaporation, hence reducing the gel thinning rate. Finally, we investigate the effect of the molecular weight of the polysaccharide on the water-holding capacity by repeating the same measurements on agarose gels of various concentrations, loaded with a mono-saccharide, glucose, instead of the agaropectin [(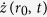) in Fig. 7(a)]. The results are identical within error bars to that obtained with the agar gel, which proves that the decrease of the thinning rate is not sensitive to the molecular weight of the polysaccharides added to the gel, but only depends on the amount of saccharides introduced inside the agarose gel.

#### Other non-gelling polysaccharide additives

To extend the aforementioned observations performed on agarose gels loaded with glucose or agaropectin, we have repeated the drying experiments with agarose gels loaded with other non-gelling saccharides of increasing molecular weights. We systematically compare the thinning rate of agarose gels loaded with 0.43% w/w of one of the following additives: glucose, dextran, guar gum or xanthan gum to the thinning rate of a pure agarose gel determined in the exact same conditions [Fig. 7(b)]. Note that such concentration corresponds to a ratio agarose/additive of 7:3, which is identical to the ratio agarose/agaropectin in agar gels. We observe that the thinning rates of loaded gels are systematically lower than that of the pure agarose gel, and that the gel thinning-rate is poorly sensitive to the molecular weight of the non-gelling additive [see Table 1 in the method section]. This result shows that, even in minute amount, non-gelling saccharides play the role of water-binding sites that efficiently slow down water evaporation and delay the shrinkage of agarose gels submitted to drying. Finally, we perform a last series of drying experiments on aqueous solutions of the same non-gelling saccharides, i.e. without any agarose, and at the same concentration (0.43% w/w). Data reported as blue open symbols in Fig. 7(b) show that the saccharides in solution slightly decrease the thinning rate of water [(★) in Fig. 7(b)], but far less than when the saccharides are embedded (at the same concentration) in an agarose gel [filled symbols in Fig. 7(b)]. These experiments demonstrate that the water-holding properties of non-gelling saccharides are strongly enhanced when embedded in a gel matrix.

## Discussion and Conclusion

In previous work recently reviewed in ref. 34, the addition of relatively large amount of sucrose to agarose gels is reported to increase the elastic modulus *G*′ and reduce the water loss, which is quantified by measuring the amount of water released (or *wept*) after an arbitrary duration (usually a few hours) from a gel submitted to an external constant load. In ref. 32, the authors explore a large range of concentrations in sucrose, always larger than 20% w/w, and show a negative correlation between the value of the elastic modulus and the extent of the water loss, which they interpret as a change in the gel microstructure for increasing sucrose content (i.e., a decrease in the pore size). Our study shows that the addition of non-gelling mono- or poly-saccharides, even in minute amounts, is enough to decrease the thinning rate of the gel without any significant change in the gel elastic modulus. Indeed, independent measurements of the elastic modulus of the agarose gels charged with non-gelling saccharides show that the amounts of additives used here do not impact the value of the elastic modulus *G*′ (see Fig. S4 in the Supplementary Information). Our study therefore proves that in the range of low concentrations, non-gelling saccharides affect the thinning rate of agarose gel without modifying their viscoelastic properties. The lower thinning rates in the presence of additives are most likely due to specific interactions such as long-lived hydrogen bonds between the water molecules and the non-gelling saccharides embedded in the agarose-gel microstructure.

To conclude, using reflection interferometry as a local investigation tool to monitor the thinning rate of agarose gels cast in Petri dishes and left to dry at constant temperature, we have shown that the gel drying kinetics is extremely sensitive to the relative heights *H* − *e*_0_ of the dish lateral walls with respect to the gel thickness. For a fixed value of *H* − *e*_0_, the thinning rate of the gel measured at the center of the dish is a robust observable that can be measured precisely through the spatiotemporal filtering method introduced in the present article and used to compare the thinning rates of gels loaded with different chemical additives. While the thinning rate of an agarose gel does not depend on the agarose concentration, the presence of agaropectin, or any other non-gelling saccharide, reduces significantly the gel thinning rate up to 40%. In a near future, we will use interferometry to determine the role of other properties of polysaccharides such as branching and hydrophilicity, on the drying of agarose gels. Finally, the approach followed in the present contribution should be useful to investigate in a systematic fashion the influence of various additives such as ions, surfactants, etc. on the drying kinetics of a wide range of polymer gels.

## Methods

### Sample preparation

Agarose-based gels are prepared as follows: hot solutions of polysaccharides are prepared by mixing either 1% w/w of agarose powder (CAS 9012-36-6, ref. A9539 Sigma-Aldrich) or 1.5% w/w of agar powder (BioMérieux, agarose/agaropectine 7:3, sulfate content 0.6% and azote content 0.45% as determined by elemental analysis) with milli-Q water (17 MΩ.cm at 25 °C) brought to a boil. Non-gelling saccharides such as glucose (CAS 50-99-7, Roquette), dextran from Leuconostoc mesenteroides (CAS 9004-54-0, Sigma Aldrich), guar gum (CAS 9000-30-0, ref. G4129 Sigma-Aldrich), and xanthan gum (CAS 11138-66-2, ref. G1253 Sigma-Aldrich) and which properties are summarized in Table 1 may also be added at this stage. The temperature is maintained constant at 100 °C for about 10 min (except for samples prepared with guar or xanthan gum that require 20 min more) and then decreased to 80 °C. The agar(ose) solution is prepared fresh for each series of experiment to avoid any aging associated with the agarose oxydation^43^,^44^. Gels, shaped as flat cylinders of (4.0 ± 0.2) mm thick are prepared by pouring the hot saccharide sol in Petri dishes of 50 mm diameter made either of smooth glass [RMS roughness of the bottom plate *R*_*q*_ = (0.53 ± 0.10) nm as determined with a Contour Elite Bruker profilometer, dish height *H* = 11 mm] or smooth polystyrene crystal (PS) [RMS roughness of the bottom plate *R*_*q*_ = (11.8 ± 3.6) nm, dish height *H* = 12 mm], and left to gelify at room temperature, i.e., T = (22 ± 2) °C. The weak adhesion of the gel to the smooth surfaces of the glass or plastic dish ensures a purely vertical shrinkage of the gel, without any sliding motion of the gel on the bottom plate of the dish, nor any detachment of the gel from the lateral wall of the dish as evidenced by the uniform and homogeneous dynamic of the local interference pattern recorded during the drying process. Finally, note that the material the Petri dish is made of has no influence on the measurements of the gel thinning rate - see Fig. S1 in the Supplementary Information. Nonetheless, smooth surfaces should be preferred. Indeed, rough surfaces lead to temporal fluctuations in the gel thinning rate due to the complex dynamics of the contact line between the gel and the lateral rough walls. See Fig. S2 in the Supplementary Information and the corresponding discussion.

## Additional Information

**How to cite this article**: Mao, B. *et al*. Impact of saccharides on the drying kinetics of agarose gels measured by *in-situ* interferometry. *Sci. Rep.* **7**, 41185; doi: 10.1038/srep41185 (2017).

**Publisher's note:** Springer Nature remains neutral with regard to jurisdictional claims in published maps and institutional affiliations.

## Supplementary Material

Supplementary Information

## Figures and Tables

**Table 1:** Properties of the non-gelling polysaccharides used as additives in agarose gels.

Additives	Formula	Molecular Weight (kDa)
Glucose	C_6_H_12_O_6_	0.18
Dextran	H(C_6_H_10_O_5_)_*x*_OH	40
*Agaropectin*	—	120 ± 30
Guar Gum	—	~220 (polydisperse)
Xanthan Gum	—	>1000

Note that agaropectin is naturally present with agarose in agar samples. To determine the molecular weight of agaropectin in the agar powder, we have separated the agaropectin from the agarose following the method described in ref. 45 and then used size exclusion chromatography^46^.
