# Supplementary material for: Impact of saccharides on the drying kinetics of agarose gels measured by in-situ interferometry
Source: Sci Rep. 2017 Jan 23;7:41185. doi: 10.1038/srep41185 (PMC5253732; doi:10.1038/srep41185)
Supplement: Supplementary Information [file srep41185-s1.pdf]

Supplemental material to: "Impact of saccharides on the drying kinetics of agarose gels measured by *in-situ* interferometry"

Bosi Mao,[1] Thibaut Divoux,[1,2] and Patrick Snabre[1]

[1]*Université de Bordeaux, Centre de Recherche Paul Pascal, CNRS UPR 8641, 115 av. Dr. Schweitzer, 33600 Pessac, France*

[2]*MultiScale Material Science for Energy and Environment, UMI 3466, CNRS-MIT, 77 Massachusetts Avenue, Cambridge, Massachusetts 02139, USA*

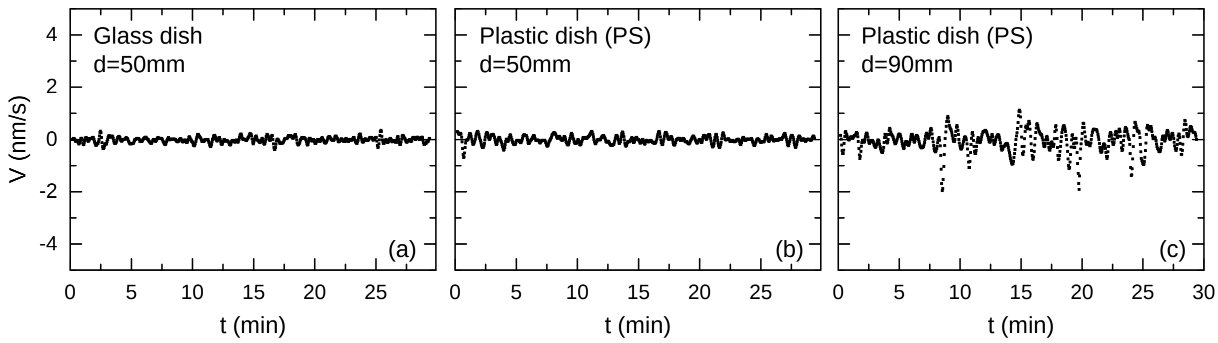

FIG. 1: Scrolling velocities of the interference fringes on the bottom plate of empty dishes vs time. Measurements performed at the center of the dish: (a) glass dish, 50 mm diameter; (b) plastic dish (PS), 50 mm diameter; (c) plastic dish (PS), 90 mm diameter. The root mean square of the velocity fluctuations in (a), (b) and (c) is respectively $\delta V = 0.1$, 0.15 and 0.5 nm/s.

Supplemental Fig.1 In order to determine the influence of the compliance of the Petri dish on the measurements of the gel thinning rate, we have performed a series of experiments on empty dishes, i.e. in the absence of any gel. The dish is placed empty in the arm of the interferometer. Changes in the interference pattern (recorded for 30 min at the center of the dish) now result from the thermal-induced deformations of the bottom plate. The same analysis as discussed in the main text is applied to the interference pattern. The thermal-induced deformation of the bottom plate are quantified for a dish of 50 mm diameter either made of glass [Fig. 1(a)] or polystyrene crystal (PS) [Fig. 1(b)], and for a 90 mm diameter dish made of polystyrene crystal (PS) [Fig. 1(c)]. The two dishes of 50 mm diameter show thermal motions of comparable amplitude, whereas the more deformable plastic dish of 90 mm diameter exhibits thermal deformations of significantly larger amplitude. We have therefore chosen to work in the main text with Petri dishes of 50 mm diameter to minimize the contribution of the dish deformability to the measurements of the gel vertical shrinkage.

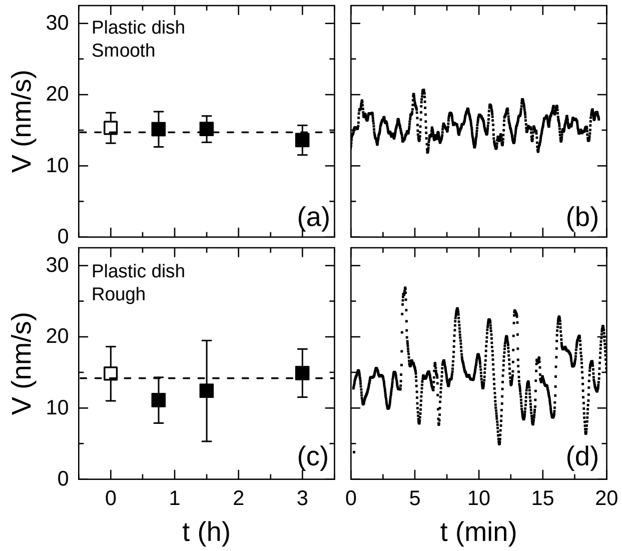

FIG. 2: Average thinning rate of a 1.5% agar gel in a smooth (a) and rough (c) plastic Petri dish PS, determined at different times during a drying experiment of 3 hours. Each point corresponds to an average over 20 min. Error bars stand for the standard deviation of the average thinning rate computed over 20 min. Horizontal dashed lines in (a) and (c) stand for the average of the data. (b) [resp. (d)] shows the time-resolved evolution of the gel thinning rate for the data associated with the first point pictured as □ in (a) [resp. (c)]. Experiments conducted at T=(25.0 ± 0.5)°C in a 50 mm diameter plastic Petri dish made of PS.

Supplemental Fig.2 In order to quantify the impact of the surface roughness of the Petri dish on the drying dynamics of the gel, we compare two drying experiments on a 1.5% agar gel casted in two different plastic dishes (PS), one with smooth boundary conditions [Mean surface roughness of (11.8 ± 3.6) nm as determined by profilometry - Fig. 2(a)], and one with rough (sand-blasted) boundary conditions [surface

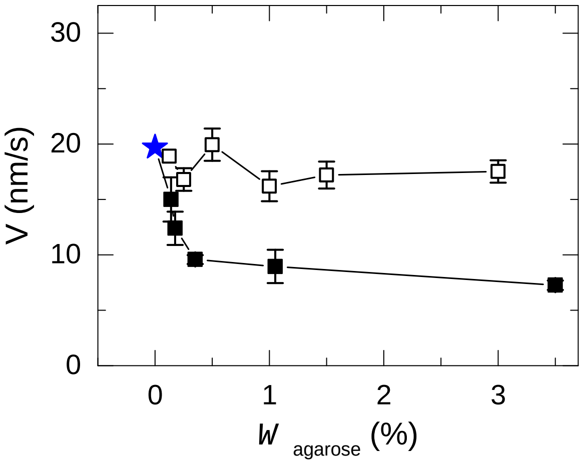

FIG. 3: (color online) Average thinning rate V of agarose (□) and agar (■) gels, determined at the center of the Petri dish, vs the concentration in agarose (% w/w). The agar employed here contains 70% of agarose. The blue star (⋆) denotes the thinning rate of a water pool with the same volume as the agar(ose) gels and monitored in the exact same experimental conditions. Error bars correspond to average standard deviation associated with three independent measurements performed over 10 minutes each. Experiments conducted at T=(20.0 ± 0.5)°C in a plastic Petri dish made of PS.

roughness of (1.54 ± 0.43) μm as determined by profilometry - Fig. 2(c)]. Over 3 hours, the average thinning rate remains constant and identical within error bars for both experiments [$\langle V_{smooth} \rangle$ =(14.8±2.1) nm/s and $\langle V_{rough} \rangle$ =(14.3±4.4) nm/s]. However, the gel thinning rate shows much larger fluctuations in the case of the rough plastic dish (PS) [Fig. 2(d) to be compared with Fig. 2(b)]. We interpret the latter result as the complex dynamics of the contact line between the gel, the lateral wall of the dish and the ambient air. The contact line may be trapped and suddenly released during the drying of the gel in a rough dish which generates a stick-slip like dynamics that impact the thinning rate.

Supplemental Fig.3 Same experiment as the one reported in Figure 7(a) in the main text. Here the drying experiments are performed at 20°C instead of 25°C. Results are shifted towards lower thinning rates, but the conclusions obtained at 25°C hold true at 20°C.

Supplemental Fig.4 We have determined the linear viscoelastic properties of agarose gels loaded with various non-gelling polysaccharides at 0.43% w/w. For each sample, the gelation is induced by a temperature ramp at 1°C/min from 70°C to 20°C in a plate-plate geometry, under constant normal force [F_N = (0.0 ± 0.1) N]. The gap follows the change in thickness of the sample as the latter goes through the sol/gel transition. See [1] for more details. The terminal values of the elastic and viscous moduli are independent of the additive and equal to that of the pure agarose gel [Fig. 4(a)]. In a similar fashion, the gelation temperature T_g of the agarose gel, defined as the intersection of the elastic and viscous moduli during the gelation process is not affected by the presence of additives [Fig. 4(b)]. Finally, the samples shrink vertically by about 1.7% during gelation independently of the nature of the additive.

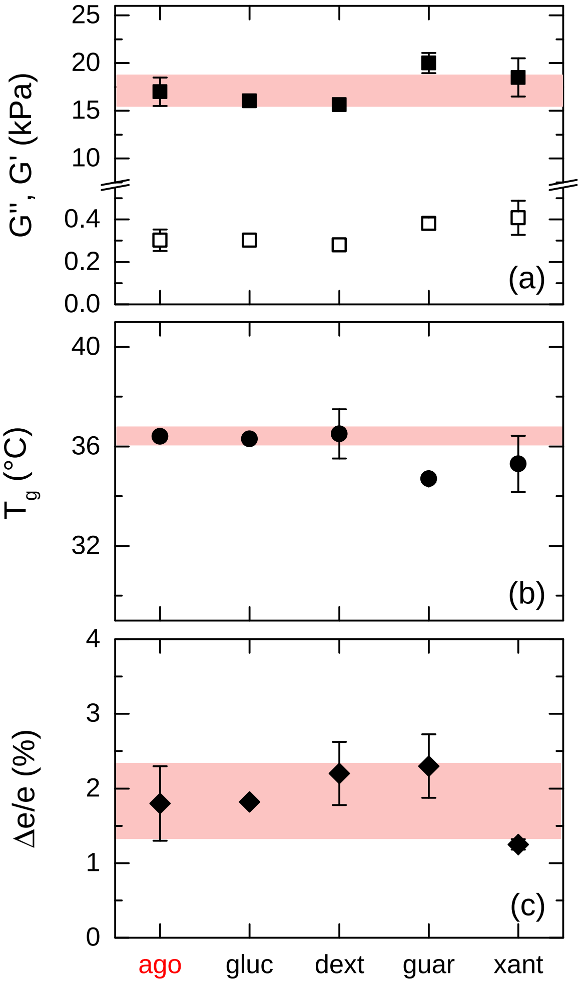

FIG. 4: (a) Elastic (■) and viscous (□) moduli of agarose gels loaded with various non-gelling polysaccharides at 0.43% w/w: none ("ago"), glucose ("gluc"), dextran ("dext"), guar gum ("guar") or xanthan gum ("xant"). (b) Gelation temperature for the same gels, as determined by the crossing of the elastic and viscous moduli during the sol/gel transition. (c) Relative contraction $\Delta e/e$ of the same gels during the gelation. Experiments performed at constant normal force [F_N = (0.0 ± 0.1) N] in a 40 mm plate-plate geometry, with an initial gap value of e_0 = 500 μm. The red stripes highlight the range of values associated with the agarose gel without any additives.

[1] B. Mao, T. Divoux and P. Snabre, Normal force controlled rheology applied to agar gelation, J. Rheol. **60**, 473–489 (2016).
